# Supplementary material for: Mechanically Robust and Flexible GO/PI Hybrid Aerogels as Highly Efficient Oil Absorbents
Source: Polymers (Basel). 2022 Nov 13;14(22):4903. doi: 10.3390/polym14224903 (PMC9696896; doi:10.3390/polym14224903)
Supplement: Supplementary file 1 [file polymers-14-04903-s001.zip › polymers-2020839-supplementary.pdf]

polymers

Supporting information

Mechanically Robust and Flexible GO/PI Hybrid Aerogels as Highly Efficient Oil Absorbents

Li Zhang, Yuting Wang *, Ruidong Wang, Penggang Yin and Juntao Wu *

Key Laboratory of Bio-Inspired Smart Interfacial Science and Technology of Ministry of Education, School of Chemistry, Beihang University, Beijing 100191, China

*Correspondence: wangyt@buaa.edu.cn (Y.W.); wjt@buaa.edu.cn (J.W.)

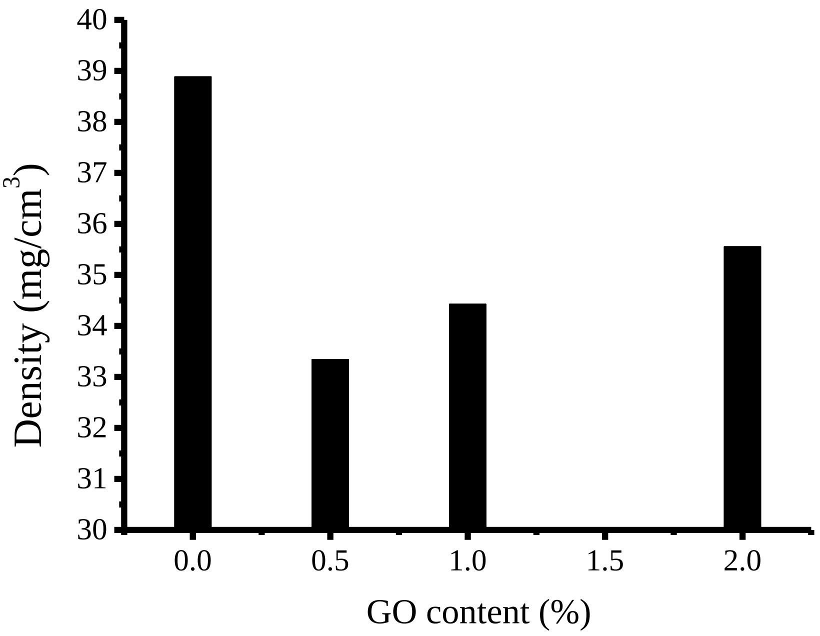

Figure S1. GIAs densities as a function of GO contents.

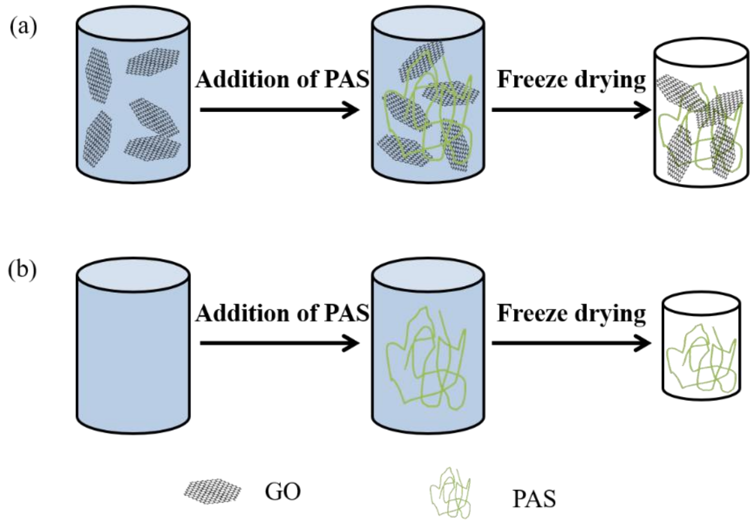

Figure S2. Schematic diagram of the freeze-drying processes of PAS solutions with GO (a) and without GO (b).

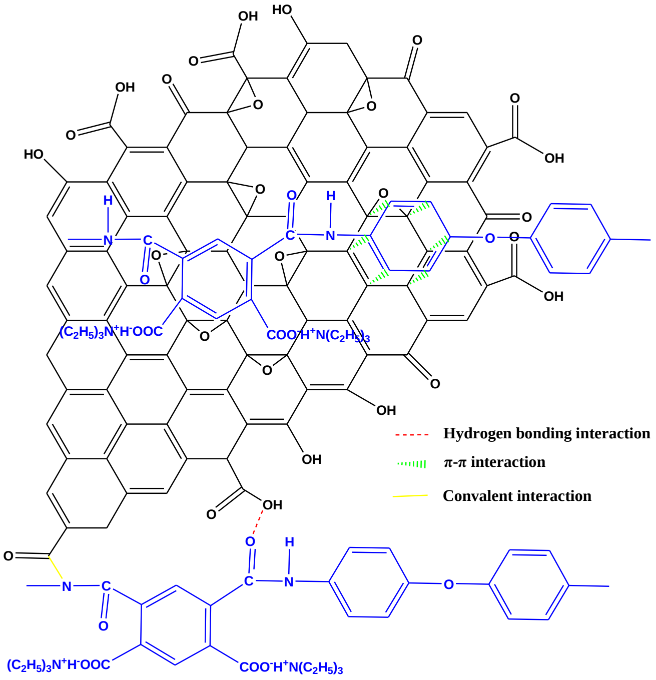

Figure S3. Schematic of the interactions that might exist between GO and PAS.

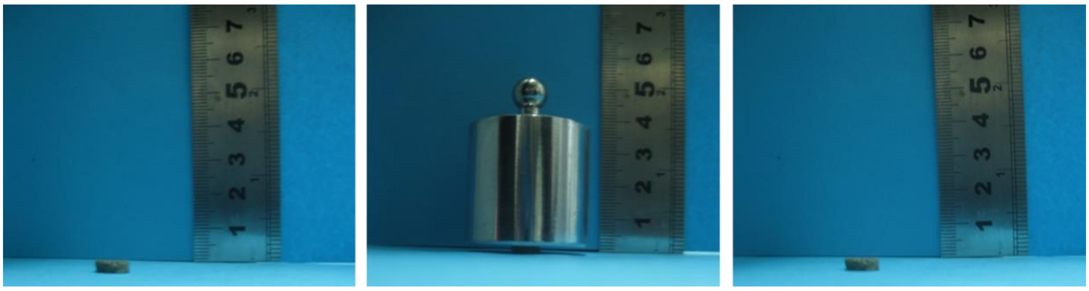

Figure S4. A GIA_1 with a weight of 6.4 mg (left); the same sample supporting a 200g poise (middle) that is 31250 times its own weight; the same sample after remove of the poise (right).

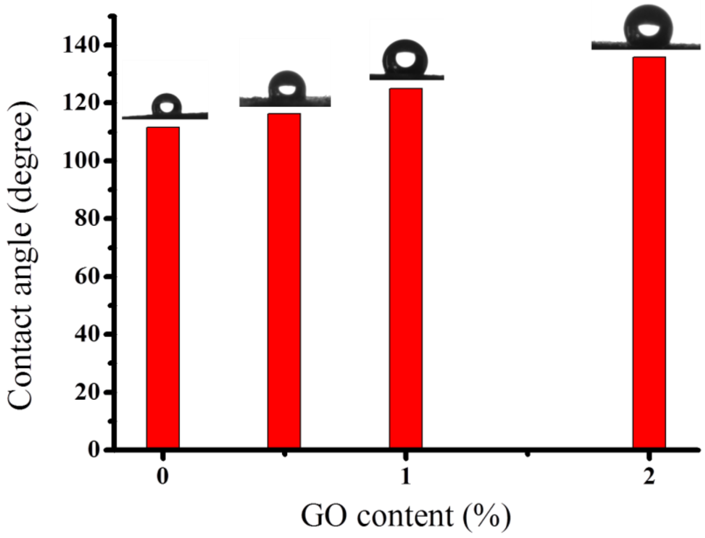

Figure S5. Water contact angles of GIAs as a function of GO contents.

Table S1. Surface tensions of organic liquid/oil absorbed by GIAs.

Organic liquid or oil	Surface tension $mN\cdot m^{-1}$	Reference
Ethanol	22.3	89
Toluene	29	89
Cyclohexane	24.3	89
Rapeseed-oil	35	90
Glycerol	63.4	91
